# Revolutionizing CRISPR technology with artificial intelligence

**DOI:** 10.1038/s12276-025-01462-9

**Published:** 2025-07-31

**Authors:** Min-gyeong Kim, Min-ji Go, Seung-Hun Kang, Soo-hwan Jeong, Kayeong Lim

**Affiliations:** 1 Biomedical Research Division, Korea Institute of Science and Technology, Seoul, Republic of Korea; 2 Division of Bio-Medical Science and Technology, KIST School, University of Science and Technology, Seoul, Republic of Korea; 3 Graduate School of Biomedical Science and Engineering, Hanyang University, Seoul, Republic of Korea

**Keywords:** Genetic engineering, Molecular engineering

## Abstract

Genome engineering has made remarkable strides, evolving from DNA-binding proteins such as zinc fingers and transcription activator-like effectors to CRISPR–Cas systems. CRISPR technology has revolutionized the field through its simplicity and ability to target specific genome regions via guide RNA and Cas proteins. Progress in CRISPR tools—CRISPR nucleases, base editors and prime editors—has expanded the toolkit to induce targeted insertions or deletions, nucleotide conversions and a wider array of genetic alterations. Nevertheless, variations in editing outcomes across cell types and unintended off-target effects still present substantial hurdles. Artificial intelligence (AI), which has seen rapid advances, provides high-level solutions to these problems. By leveraging large datasets from diverse experiments, AI enhances guide RNA design, predicts off-target activities and improves editing efficiency. In addition, AI aids in discovering and designing novel CRISPR systems beyond natural limitations. These developments provide new modalities essential for the innovation of personalized therapies and help to ensure efficiency, precision and safety. Here we discuss the transformative role of AI in advancing CRISPR technology. We highlight how AI contributes to refining nuclease-based editing, base editing and prime editing. Integrating AI with CRISPR technology enhances existing tools and opens doors to next-generation medicine for gene therapy.

## Enhancing CRISPR tools with artificial intelligence innovations

Early methods of genome editing used complex proteins, but the discovery of CRISPR–Cas9 simplified the process. CRISPR–Cas9 uses a guide RNA to direct the Cas9 protein to specific DNA sites for precise editing. However, CRISPR can sometimes make mistakes. To improve accuracy, researchers are using artificial intelligence (AI) to enhance CRISPR technology. AI is helping scientists to design better guide RNAs and predict potential errors in CRISPR editing. This involves using machine learning to analyze large datasets and improve the precision of genome editing tools. The integration of AI has led to more accurate predictions of CRISPR’s effects and has helped in designing new proteins for genome editing. These advancements make CRISPR efficient and safer for use in research and potential medical applications. In conclusion, AI is substantially enhancing CRISPR technology by improving its precision and efficiency.

This summary was initially drafted using artificial intelligence, then revised and fact-checked by the author.

## Introduction

Early genome engineering was developed on the basis of DNA-binding proteins such as zinc-finger proteins^1,2^ and transcription activator-like effector arrays^3–5^. Protein-based genome editing technologies have worked well so far with their unique advantages, but their design often involves a time-consuming process of protein engineering^6–8^. The later discovery of the CRISPR–Cas system^9–12^ brought a fundamental turn by providing a simplified and versatile method for targeted gene perturbation^13^. Among the various CRISPR applications, the CRISPR–Cas9 system has become the most widely used, using a guide RNA (gRNA) to navigate the Cas9 protein to a specific genomic locus for precise DNA cleavage. This breakthrough has expanded the possibilities of genetic engineering and positioned CRISPR as a key tool across multiple disciplines, including genetics, molecular biology and biomedicine.

**Fig. 1:**
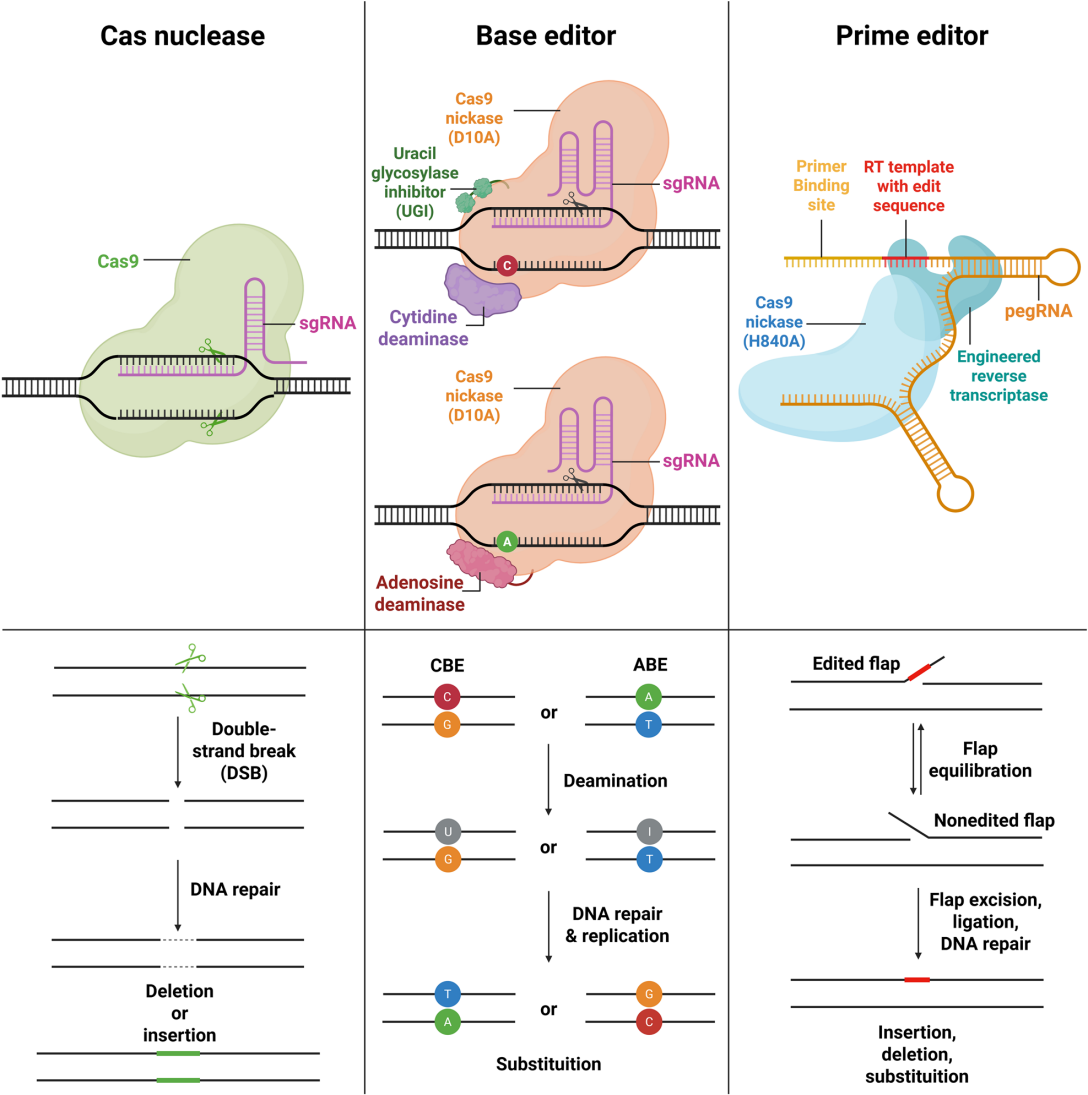
CRISPR-based genome editing tools. CRISPR-based genome editing tools include CRISPR nucleases, base editors and prime editors. Cas nucleases are composed of the Cas9 protein and single guide RNA (sgRNA), which induce DSBs. CRISPR nucleases can be used for insertions, deletions and point mutations, as well as enabling chromosomal translocations. Base editors are composed of a catalytically impaired Cas9, sgRNA and a deaminase. Base editors primarily mediate C-to-T or A-to-G conversions without generating DSBs. Prime editors consist of a Cas9 nickase, an engineered reverse transcriptase and pegRNA, which contains a PBS and reverse transcription (RT) template encoding the desired edit. Prime editors are capable of small insertions and deletions and can facilitate all types of point mutation. The editing efficiency of each tool can be enhanced through AI-driven approaches.

As CRISPR technology evolves, its applications extend beyond the original CRISPR–Cas9 system to various platforms for specific genetic changes^14–16^. There are currently three CRISPR-derived DNA editing technologies that have entered clinical trials^17^: Cas nucleases, base editors and prime editors (Fig. 1). CRISPR nucleases, such as Cas9 (refs.^9–12^) and its engineered variants, induce double-strand breaks (DSBs) that trigger DNA repair, resulting in insertions or deletions (indels). However, unintended indels caused by inaccurate nuclease activity of CRISPR pose risks of permanent damage, which limits precision in applications. To overcome this concern, alternative technologies have emerged, incorporating effector domains that enable desired changes without the direct action of Cas nucleases. Base editing, for example, combines deaminases with catalytically defective Cas proteins to precisely convert C-to-T or A-to-G nucleotides^18–20^. Prime editing, a more refined technique, supports a wider range of genetic modifications—including insertions, deletions and all other types of substitution—by fusing an engineered reverse transcriptase and an extended gRNA^21^ (Fig. 1). In addition to DNA editing technologies, other CRISPR-based tools have been devised that utilize RNA-modifying enzymes for RNA editing, transcriptional effectors for gene regulation and epigenomic modifiers for epigenome editing^14^.

Despite this remarkable progress, challenges still exist with CRISPR technology: variable efficiencies across cell types, dependence on sequence context for on-target activities and frequent off-target activities throughout the genome. To address these issues, advanced computational approaches are increasingly being used to optimize CRISPR systems^22^. Artificial intelligence (AI), particularly machine learning (ML), has become indispensable in refining gRNA design, predicting off-target effects and improving editing efficiency through the analysis of large datasets from diverse experiments. AI-driven models are effective in enhancing current CRISPR technologies, as well as guiding the development of cutting-edge tools. In this Review, we will explore how AI is revolutionizing CRISPR technology, focusing on improving CRISPR nuclease, base editing and prime editing for more precise and scalable genome-editing applications.

## AI-driven innovation in genome engineering

AI enables computers to perform tasks that typically require human intelligence. A subset of AI, ML, involves algorithms that learn from data to identify patterns and make predictions or decisions^23^. It encompasses various approaches (Table 1), including supervised learning, unsupervised learning and reinforcement learning. Deep learning (DL)^24^, a specialized area within ML, leverages artificial neural networks and supports various learning approaches, making it a powerful tool for processing complex data.

**Table 1:** AI models used in this Review and their acronyms.

Acronym	Definition
AttnBiRNN	Attention-based bidirectional recurrent neural network
BiLSTM	Bidirectional long short-term memory neural network
CNN	Convolutional neural network
CRNN	Convolutional recurrent neural network
DCAR	Deep conditional autoregressive model
DCDNN	Deep convolutional denoising neural network
GBM	Gradient boosting machine
GBR(T)	Gradient-boosted regression tree
k-NN	*k*-Nearest neighbors
LightGBM	Light gradient boosting machine
LRCN	Long-term recurrent convolutional network
MLP	Multilayer perceptron
RF	Random forest
RNN	Recurrent neural network
SVM	Support vector machine
XGBoost	Extreme gradient boosting

In ML^25^, supervised learning is a common approach in which a model is trained on a labeled dataset, with each training example paired with an output label. This method is effective when input data and corresponding labels are available, enabling the model to learn a function that generates the correct outputs based on the input data. Alternatively, unsupervised learning processes unlabeled data, allowing the model to identify hidden patterns. This often involves clustering data points to identify similarities or characteristics. Reinforcement learning^26^ is a method of interacting with an environment, taking specific actions and receiving feedback (rewards) based on the outcomes, gradually learning to maximize these rewards through repeated interactions. Unlike traditional ML methods, DL can be applied across supervised, unsupervised and reinforcement learning, making it especially versatile and effective in processing large and complex datasets.

Building on the foundational principles of DL, generative AI, including language models, has emerged as a powerful tool for generating new content and structures by learning from existing data. Recently developed DL technologies have enabled the prediction of protein structures based on amino acid sequences. Unlike traditional physics-based models, AI-driven approaches derive insights from large-scale data^27^. In 2020, Google DeepMind introduced AlphaFold1 (ref.^28^), marking the beginning of DL-based protein structure prediction. The subsequent development of AlphaFold2 (ref.^29^) in 2021 achieved near-experimental accuracy. Around the same time, the Baker laboratory introduced RoseTTAFold^30^, another DL-based protein structure prediction model. After that, models such as RoseTTAFoldNA^31^ and RoseTTAFold All-Atom^32^ were developed to extend structure prediction capabilities to nucleic acids and atomic-level modeling, respectively. More recently, AlphaFold3 (ref. ^33^) was developed, integrating generative AI models to extend beyond protein structure prediction, enabling the modeling of interactions with nucleic acids and other biomolecules. The development of AI-driven protein structure prediction models has profoundly transformed structural biology and was recognized with the 2024 Nobel Prize in Chemistry. These systems illustrate the broad potential of AI-driven models, with applications spanning fields from language processing to biological research.

The integration of AI technologies has addressed key limitations in conventional CRISPR technology, thereby enhancing genome editing with greater precision and efficiency. In particular, AI-based models are valuable in designing optimal gRNAs and predicting off-target effects from large genomic datasets, notably improving genome editing performance. By automating and optimizing processes previously performed manually, AI allows researchers to focus on solving more complex problems. Moreover, beyond simple data analysis, AI-driven models generate novel DNA, RNA and amino acid sequences^34,35^, bringing innovation to genome editing fields that were difficult to access. The following sections discuss the contribution of AI to the advancement of CRISPR technology, drawing from relevant research articles.

## Enhancing CRISPR–Cas nucleases with AI

The CRISPR–Cas nuclease system is an RNA-guided endonuclease originally identified in bacterial immune systems^9^. It stores information about the nucleic acids of previously invading pathogens to cut them if they invade again. The CRISPR–Cas system can be divided into two parts: the Cas protein, which binds and cleaves DNA, and the gRNA, which directs the Cas protein to specific target DNA (Fig. 1). Due to its relatively easy design, the CRISPR–Cas system has become a powerful tool for genome editing. However, it can induce off-target effects, and DNA repair mechanisms against Cas-mediated cleavage are difficult to predict^36^.

### Optimizing gRNA design for high activity in the CRISPR–Cas system

**Fig. 2:**
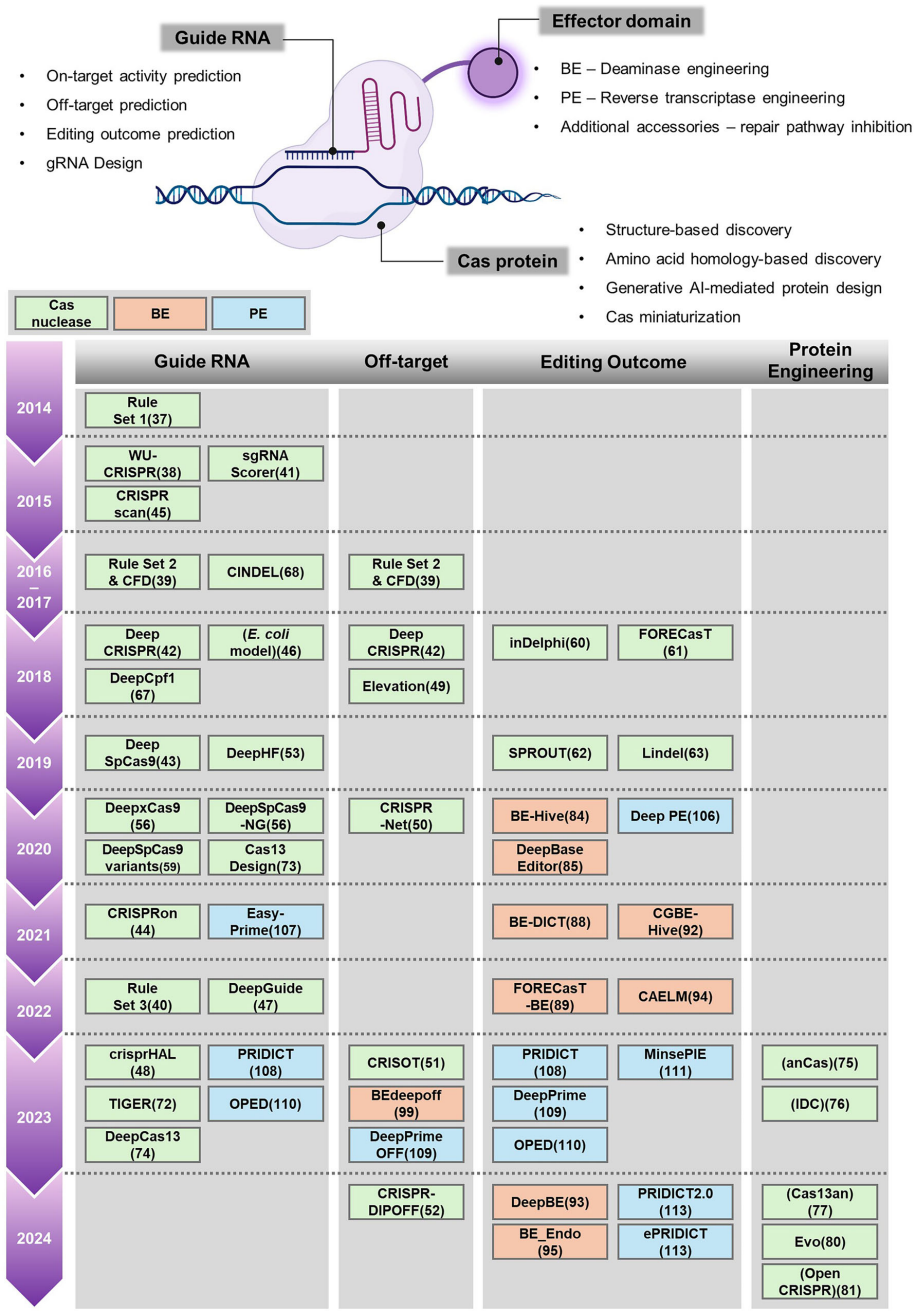
AI-driven prediction and engineering in CRISPR systems. The timeline illustrates AI-assisted advancements in CRISPR-based genome editing, categorizing developments in gRNA design, off-target prediction, editing outcome prediction and protein engineering. ML and DL models are applied to predict the on-target and off-target activities of gRNA, along with editing efficiency and patterns. These models also facilitate the design of optimized gRNAs. Furthermore, AI-driven protein design can be utilized for protein engineering. Advanced tools such as AlphaFold3 enable structure-based discovery, amino acid homology-based exploration and Cas miniaturization. The color-coded classification differentiates models targeting Cas nucleases (in green), base editors (BE, in orange) and prime editors (PE, in blue), highlighting their specific contributions to genome engineering. Through these advancements, AI continues to refine CRISPR technologies, enhancing precision and broadening their applications in genome engineering.

Experiments using the CRISPR–Cas system can vary considerably depending on the gRNA used. Therefore, knowing the activity of gRNA in advance can greatly enhance the success rate of experiments. Accordingly, several AI-based research models have been developed to predict gRNA activity (Fig. 2 and Table 2).

**Table 2:** CRISPR–Cas nuclease system-associated AI prediction models.

Name	CRISPR system	Organism	AI model used	Purpose	Reference (year)	Web tool or code link
Rule Set 1	SpCas9	Human, mouse	SVM, logistic regression	gRNA prediction	^37^ (2014)	(Update to CRISPick)
WU-CRISPR	SpCas9	Human, mouse	SVM	gRNA prediction	^38^ (2015)	http://crisprdb.org/wu-crispr/
Rule Set 2 and CFD	SpCas9	Human, mouse	GBRT	gRNA prediction, off-target prediction	^39^ (2016)	(Update to CRISPick)
Rule Set 3 (CRISPick)	SpCas9	Human	LightGBM	gRNA prediction	^40^ (2022)	https://portals.broadinstitute.org/gppx/crispick/public
sgRNAScorer	SpCas9, St1Cas9	Human	SVM	gRNA prediction	^41^ (2015)	http://crispr.med.harvard.edu/sgRNAScorer
DeepCRISPR	SpCas9	Human	DCDNN, CNN	gRNA prediction, off-target prediction	^42^ (2018)	https://github.com/bm2-lab/DeepCRISPR
DeepSpCas9	SpCas9	Human	CNN	gRNA prediction	^43^ (2019)	http://deepcrispr.info/DeepSpCas9
CRISPRon	SpCas9	Human	GBRT, CNN	gRNA prediction	^44^ (2021)	https://rth.dk/resources/crispr/
CRISPRscan	SpCas9	Zebrafish	Logistic regression, Linear regression	gRNA prediction	^45^ (2015)	http://CRISPRscan.org
(None) (*E. coli* model)	SpCas9, eSpCas9, SpCas9(Δ recA)	*E. coli*	GBRT	gRNA prediction	^46^ (2018)	https://github.com/zhangchonglab/sgRNA-cleavage-activity-prediction.git
DeepGuide	SpCas9, LbCas12a	Yeast	CNN	gRNA prediction	^47^ (2022)	https://github.com/dDipankar/DeepGuide
crisprHAL	SpCas9, TevSpCas9	*E. coli*	CNN, RNN	gRNA prediction	^48^ (2023)	https://github.com/tbrowne5/crisprHAL
Elevation	SpCas9	Human	Boosted regression tree, L1-regularized linear regression, logistic regression, gradient-boosted regression tree	Off-target prediction	^49^ (2018)	https://crispr.ml
CRISPR-Net	SpCas9	Human	LRCN	Off-target prediction	^50^ (2020)	https://codeocean.com/capsule/9553651/tree/v1
CRISOT	SpCas9	Human	XGBoost	Off-target prediction	^51^ (2023)	https://github.com/bm2-lab/CRISOT
CRISPR-DIPOFF	SpCas9	Human	RNN (LSTM)	Off-target prediction	^52^ (2024)	https://github.com/tzpranto/CRISPR-DIPOFF
DeepHF	eSpCas9(1.1), SpCas9-HF1	Human	RNN (BiLSTM)	gRNA prediction	^53^ (2019)	http://www.DeepHF.com/
DeepxCas9, DeepSpCas9-NG	xSpCas9, SpCas9-NG	Human	CNN	gRNA prediction	^56^ (2020)	http://deepcrispr.info/DeepxCas9 http://deepcrispr.info/DeepSpCas9-NG
DeepSpCas9variants	13 SpCas9 variants	Human	CNN	gRNA prediction	^59^ (2020)	http://deepcrispr.info/DeepSpCas9variants
inDelphi	SpCas9	Human, mouse	MH-NN (Microhomology-Neural Network), MHless-NN	Editing outcome pattern prediction	^60^ (2018)	https://indelphi.giffordlab.mit.edu/
FORECasT	SpCas9	Human, mouse	Logistic regression	Editing outcome pattern prediction	^61^ (2018)	https://partslab.sanger.ac.uk/FORECasT
SPROUT	SpCas9	Human	Gradient boosting	Editing outcome pattern prediction	^62^ (2019)	https://zou-group.github.io/SPROUT
Lindel	SpCas9	Human	Logistic regression	Editing outcome pattern prediction	^63^ (2019)	https://lindel.gs.washington.edu
CINDEL	AsCas12a	Human	Logistic regression	gRNA prediction	^68^ (2017)	(Update to deepcpf1)
DeepCpf1	AsCas12a	Human	CNN	gRNA prediction	^67^ (2018)	http://deepcrispr.info
Cas13design	RfxCas13d	Human	RF	gRNA prediction	^73^ (2020)	https://cas13design.nygenome.org
DeepCas13	RfxCas13d	Human	CRNN	gRNA prediction	^74^ (2023)	http://deepcas13.weililab.org
TIGER	RfxCas13d	Human	CNN	gRNA prediction	^72^ (2023)	https://tiger.nygenome.org/

First, Doench et al.^37^ assembled tiling gRNA pools for six and three endogenous genes in mice and humans, respectively, and evaluated their ability to generate null alleles through SpCas9. They classified the top 20% of gRNAs with high activity, investigated their sequence features and applied this information to develop the gRNA activity prediction model, Rule Set 1. Wong et al.^38^ utilized the previous dataset^37^ to classify and compare the top 20% and bottom 20% of gRNAs, identifying structural and sequence features to develop an improved gRNA activity prediction model. Subsequently, Doench et al.^39^ constructed a human/mouse genome-targeting gRNA library and leveraged it to establish the on-target activity prediction model, Rule Set 2. They also conducted screening with a library that included not only perfectly matched gRNAs but also those with insertions, deletions or mismatches, which led to the derivation of the cutting frequency determination (CFD) score for off-target activity prediction. Following this, Rule Set 3 (ref. ^40^) elucidated how variations among *trans*-activating CRISPR RNA (tracrRNA) variants could influence gRNA activity, incorporating these insights into a prediction model using tools such as light gradient boosting machine (LightGBM).

To build a reliable prediction model, it is crucial to either obtain a sufficient quantity of data or to secure high-quality data. To this end, some studies have utilized available datasets, while others have improved screening platforms. Chari et al. generated an in vivo library-on-library methodology, which involved lentiviral integration of targets and conducting two rounds of testing, to evaluate multiple gRNAs across approximately 1,400 genomic loci. Using screening results from multiple human cell lines with SpCas9 and St1Cas9, they created sgRNAScorer^41^, a tool for predicting gRNA activity. In a separate case, Chuai et al. formulated DeepCRISPR^42^, a DL model that utilizes gRNA data with known on-target efficacy and off-target profiles. The model predicts both on-target efficiencies and genome-wide off-target effects of Cas9 simultaneously. This study addressed data imbalances through augmentation and bootstrapping to enhance model performance. Additionally, Kim et al. performed a high-throughput screening of 12,832 target sequences in human cells using a library that included the target DNA and the corresponding gRNA. They designed the activity prediction model DeepSpCas9 (ref. ^43^) using a convolutional neural network (CNN), which showed better generalization across different datasets compared with existing models. Another large dataset of 23,902 gRNAs led to the creation of CRISPRon^44^, an efficiency prediction model for gRNAs. This study also confirmed that the binding energy between gRNA and DNA is a key factor in feature analysis.

In addition to human and mouse cell lines, researchers have also developed prediction models based on data from other species and cell lines. For instance, a study on zebrafish embryos tested 1280 gRNAs targeting 128 genes, as well as 640 alternative gRNAs, including truncated, extended and 5′-mismatched variants. Based on the screening results, the prediction model CRISPRscan^45^ was created using logistic and linear regression to analyze sequence features. This research identified several determinants, such as guanine enrichment and adenine depletion in gRNA activity. A distinct study conducted a genome-scale screening in *Escherichia coli* using a library of approximately 70,000 gRNAs to develop a gRNA prediction model for Cas9 and its variants^46^. This study is expected to aid in designing new antimicrobial agents.

Because existing prediction models were tailored to the organism from which the training data were derived, a platform capable of designing gRNAs specific to individual organisms was required. In response, Deepguide^47^ was designed using gRNA activity profiles for SpCas9 and LbCas12a in *Yarrowia lipolytica* and was used to predict high-activity gRNAs on the basis of this dataset. This model can be applied to various other species through retraining. An additional study used a two-plasmid positive selection system in *E. coli*, which included a toxin expression plasmid that allowed only cells with the corresponding toxin removed by on-target activity to survive. Using these data, the prediction model crisprHAL was developed^48^, which enables accurate predictions and can be applied to other bacteria.

### Reducing off-target effects in the CRISPR–Cas system

The term off-target effect refers to the unintended activity of genome engineering tools at sites other than the intended target. While on-target activity is certainly important, if the off-target effects are severe, severe off-target effects may make it challenging to apply this system. Thus, predicting off-target activity is essential for enhancing the safety and specificity of the CRISPR–Cas system (Fig. 2 and Table 2). Several studies has focused on addressing this issue through better prediction methods.

Listgarten et al.^49^ developed a model named Elevation for off-target prediction, which performs gRNA–target pair scoring and gRNA summary scoring. In a parallel study, Lin et al. utilized data from previous research to create CRISPR-Net^50^, a model that predicts gRNA off-target activity through a long-term recurrent convolutional network (LRCN). Another study^51^ focused on analyzing the molecular interactions between RNA and DNA in the CRISPR system through simulations. This research collected various genome-wide off-target datasets and trained an extreme gradient boosting (XGBoost) classification model to calculate off-target scores and gRNA specificity scores^51^. By contrast, Toufikuzzaman et al.^52^ formulated CRISPR-DIPOFF, an off-target prediction model leveraging recurrent neural networks (RNNs) on the datasets utilized in DeepCRISPR^42^. CRISPR-DIPOFF addresses the precision–recall trade-off, a common limitation in conventional off-target prediction models, by using RNN variants such as vanilla RNN, long short-term memory (LSTM) and gated recurrent unit (GRU) to enhance the balance between precision and recall. Among these, LSTM outperformed the others and was selected as the final model for CRISPR-DIPOFF.

### Improving performance with Cas variants

Numerous variants of SpCas9 with enhanced activity and specificity have emerged, prompting the development of prediction models for these engineered variants. One of the first models, DeepHF^53^, was developed to predict gRNA activity based on genome-scale screening results in human cells, focusing on the high-fidelity versions of SpCas9, such as eSpCas9(1.1)^54^ and SpCas9-HF1^55^. After that, Kim et al.^56^ conducted high-throughput screening, targeting more than 26,000 lentiviral integration sites and 78 endogenous sites in human cells to evaluate and compare the protospacer adjacent motif (PAM) compatibility of SpCas9, xCas9^57^ and SpCas9-NG^58^. Based on these results, a DL model was formulated to predict on-target and off-target activities of Cas9 variants. Furthermore, a model that can predict and compare the activity of various Cas9 variants on target sequences was created using screening data from 13 SpCas9 variants across 26,891 target sequences^59^, facilitating the selection of the appropriate platform.

### Refining editing outcomes and mutation patterns in the CRISPR–Cas system

The ability to predict the pattern of editing outcomes before designing an experiment can effectively enhance the chances of achieving the desired results (Fig. 2 and Table 2). First, Shen et al. conducted a screening to characterize the repair products after template-free Cas9 cleavage in five human and mouse cell lines. From these results, they designed inDelphi, a model to predict genotypes and frequencies of 1–60-bp deletions and 1-bp insertions^60^. They found that certain gRNAs could guide the repair process to produce a single genotype in at least 50% of editing products. In addition, they pinpointed human pathogenic alleles that could be corrected without a template and achieved over 50% correction in practice. In a different study, Allen et al. used a library containing both the gRNA and the target sequence, with variable contextual configurations around the target sequence, to screen over 40,000 gRNAs and examine the repair outcomes. They found that the repair results are biased and local sequence dependent, leading to the development of FORECasT^61^. This research also revealed that repair tendencies vary across cell lines. Similarly, Leenay et al. developed a model called SPROUT^62^ using the repair outcomes of Cas9-mediated cleavage at >1600 target sites in primary T cells obtained from 18 individuals. The model demonstrated higher accuracy than inDelphi and FORECasT when tested on data from other primary T cells and induced pluripotent stem cells. In another study, mutations resulting from Cas9 and repaired patterns were profiled for over 6000 targets in human cells^63^. With this dataset, Lindel was generated to predict mutation outcomes considering the local sequence context. This model exhibited greater accuracy in predicting the ratio of insertions to deletions compared with FORECasT.

### Expanding applications to other CRISPR systems

In addition to the Cas9 system, other systems have been discovered^64^, and considerable research has been conducted on predictive models to predict their functionality. Unlike the Cas9 system, which requires an RNA composed of CRISPR RNA (crRNA) and tracrRNA, the Cas12a (Cpf1) system needs only crRNA^65,66^. For AsCas12a (AsCpf1), a CNN-based DL model called DeepCpf1 (ref. ^67^) was developed. This model incorporates chromatin accessibility information to enhance performance and can accurately predict AsCas12a activity in 125 cell lines. Compared with an earlier classification model, CINDEL^68^, DeepCpf1 notably improves the accuracy of activity predictions. Both models applied libraries containing the target sequence and corresponding gRNA, enabling the evaluation of Cas12a activity in a high-throughput manner.

Unlike the CRISPR–Cas9 and CRISPR–Cas12 system, which target DNA, the CRISPR–Cas13 system targets RNA^69–71^. TIGER^72^, a DL model based on CNN, was designed for RfxCas13d (CasRx)^71^. This model outperformed earlier models, Cas13design^73^ and DeepCas13^74^, that used a random forest (RF) model and a convolutional recurrent neural network (CRNN)-based model when compared across four evaluation metrics (Pearson, Spearman, AUROC (area under the receiver operating characteristic curve) and AUPRC (area under the precision-recall curve)). The study found that the model can predict the efficacy for both perfect-match and mismatched gRNAs and that such mismatches can affect Cas13d activity.

### Generating Cas proteins and discovering new proteins

The research on Cas proteins has been as comprehensive as studies involving gRNA or related sequences (Fig. 2). Alonso-Lerma et al.^75^ investigated the origins of the Cas9 protein using ancestral sequence reconstruction techniques to resurrect the ancestors of Cas9 (anCas). They selected five asCas9 proteins and then utilized the DL model AlphFold2 to predict their structure. Through deep structural analysis and activity profiling of these asCas9s, they uncovered the functional evolutionary trajectory of Cas9. Furthermore, Zhao et al.^76^ devised an interaction, dynamics and conservation (IDC) strategy to minimize the protein size using structural information such as nucleoprotein interaction, dynamic conformation reorganization and ortholog conservation. The authors utilized AlphaFold2 to predict the structure of the Cas13 protein, and developed five miniaturized variants by using the IDC strategy, which effectively minimizes protein size while maintaining enzymatic structure by removing unnecessary regions. In a different study, Yoon et al.^77^ created a structure homology-based search pipeline by leveraging a Foldseek-clustered AlphaFold database^78,79^. They used two representative HEPN domains (HEPN1 and HEPN2) of Cas13 as search queries and identified a novel ancestral clade of Cas13 (Cas13an). Cas13an, one-third the size of other Cas13 proteins, exhibited strong RNA depletion and effective antiphage defense in *E. coli*.

Moreover, a generative model named Evo^80^ was devised to predict and generate biological sequences ranging from the molecular level to a genome-wide scale. Evo was trained on over 80,000 genome and metagenome datasets from prokaryotic genome and phage genomes. It successfully predicted functions across DNA, RNA and proteins, as well as gene essentiality at nucleotide resolution. Evo also generated novel CRISPR–Cas complexes along with their gRNAs, transposable elements and genome-scale coding sequences of approximately 650 kilobases. In a separate study, Ruffolo et al.^81^ adjusted a language model, like ProGen2 (ref. ^82^), to generate multiple new proteins. They utilized the CRISPR–Cas Atlas, a dataset comprising 1,246,163 CRISPR operons, to create millions of novel CRISPR–Cas sequences. In addition, they selected 238,917 Cas9 sequences from the CRISPR–Cas Atlas to train the model and generate Cas9-like sequences. From the generated Cas9-like sequences, 209 were tested for functionality in human cells, and the top hit, OpenCRISPR-1, was identified. Despite having lower sequence similarity to SpCas9, OpenCRISPR-1 exhibited increased activity. Importantly, this study represents the first successful instance of human genome editing using an entirely AI-designed complex, highlighting the transformative potential of AI-driven gene-editing technologies.

## Advancing CRISPR base editing with AI

The most well-known base editors are cytosine base editors (CBEs)^50^ and adenine base editors (ABEs)^18^ that mediate C-to-T and A-to-G substitutions, respectively (Fig. 1). Base editors were engineered by fusing a deaminase protein to a catalytically impaired Cas protein, which retains its binding activity, enabling precise single-base substitution without generating DSBs. Deaminases are base-modifying enzymes that act on single-strand DNA (ssDNA). After gRNA-dependent CRISPR targeting, the CRISPR complex unwinds the target DNA, exposing a short ssDNA segment. The cytosine or adenine deaminase then catalyzes the conversion of cytosine or adenine within exposed ssDNA, transforming the deaminated cytosine to thymine or adenine to guanine through DNA repair or replication mechanisms. Base editors show higher editing efficiency with low indel frequencies^83^; however, they can cause bystander editing near the target base and have the limitation that they cannot edit all kinds of point mutation.

### Improving the efficiency of base editors

Base editors possess substantial potential as genome editing tools for fundamental research and gene therapy. Yet, their application has been restricted by the considerable variability in editing performance across target sites. With the help of AI technology, several research teams have sought to address this issue by developing predictive models for base editing efficiency (Fig. 2 and Table 3).

**Table 3:** Base editor system-associated AI prediction model.

Name	Base editing system	Organism	AI-model used	Purpose	Reference (year)	Web tool or code link
BE-Hive	2 ABEs, 6 CBEs	Human, mouse	Logistic regression, GBRT, DCAR	Base editing outcome prediction	^84^ (2020)	www.crisprbehive.design
DeepBaseEditor	ABE7.10, BE4	Human	CNN	Base editing outcome prediction	^85^ (2020)	https://deepcrispr.info/DeepBaseEditor/
BE-DICT	ABEmax, CBE4max, ABE8e, Target-AID	Human	Attention-based neural network	Base editing outcome prediction	^88^ (2021)	www.be-dict.org.
FORECasT-BE	BE4,ABE8e, ABE8.20-m	Human	GBM	Base editing outcome prediction	^89^ (2022)	https://partslab.sanger.ac.uk/FORECasT-BE
CGBE-Hive	10 CGBEs	Human, mouse	Logistic regression, GBRT, DCAR	Base editing outcome prediction	^92^ (2021)	www.crisprbehive.design
DeepBE	63 BEs	Human	CNN	Base editing outcome prediction	^93^ (2024)	https://deepcrispr.info/DeepBE/
CAELM	BE4max	Human	GBRT (XGBoost)	Base editing outcome prediction	^94^ (2022)	https://github.com/YQLiCAS/BE4max
BE_Endo	ABEmax, BE3-FNLS	Human	CNN	Base editing outcome prediction	^95^ (2024)	http://www.sunlab.fun:3838/BE_Endo
BEdeepoff	AncBE4max, ABEmax	Human	RNN (BiLSTM)	Off-target prediction	^99^ (2023)	http://www.deephf.com/#/bedeep/bedeepoff

Through the analysis of a dataset from 38,538 target sequences in mammalian cells, Arbab et al. generated a ML model called BE-Hive^84^, characterizing the relationship between sequence context and the activity of CBE and ABE. They used BE-Hive to correct over 3000 disease-associated and pathogenic transversion variants (SNVs) and 174 pathogenic transversion SNVs with high precision. Furthermore, they developed new CBE variants, such as EA-BE4 and eA3A-BE5, providing narrow-window base editing. EA-BE4 (BE4 H47E + S48A) is a precise base editor that reduces undesired cytosine-to-guanine transversions while preserving the editing properties of the original BE4. eA3A-BE5 (eA3A-BE4 T44D + S45A) enhances single-nucleotide precision and reduces byproducts without sacrificing efficiency. In another investigation, Song et al. evaluated the efficiency of BE4 (rAPOBEC1-CBE) and ABE7.10^18^ (ecTadA7.10-ABE) in over 13,000 target sequences in human cells and created DeepBaseEditor (DeepCBE and DeepABE)^85^, a DL-based computational model. They applied this tool to predict the efficiency and outcome frequencies of CBE and ABE in target sequences. In a separate study, Marquart et al. performed an extensive analysis of ABEmax (ecTadA7.10-ABE)^86^, BE4max (rAPOBEC1-CBE)^86^, ABE8e (ecTadA8e-ABE)^87^ and Target-AID (PmCDA1-CBE)^20^ using a large lentiviral library containing gRNA-expressing cassette and target DNA^88^. They formulated an attention-based DL algorithm called BE-DICT, which can predict base editing results with remarkable accuracy. The authors conducted a comprehensive comparison of BE-Hive, DeepBaseEditor and BE-DICT, finding similar levels of accuracy. However, unlike the other models, BE-DICT offers a per-base module that identifies highly preferred or disfavored motifs in existing base editors, enabling the prediction of new variants with enhanced activity. Similarly, Pallaseni et al.^89^ measured the editing frequencies of FNLS and BE4GamRA for CBE (engineered rAPOBEC1-CBE), and ABE8e and ABE8.20-m for ABE (engineered ecTadA7.10-ABE)^87^, across approximately 14,000 target sequences in a human cell line and discovered a new sequence bias that considerably impacts the editing efficiency at specific positions. Based on these findings, they developed FORECasT-BE, a ML model for predicting per-position editing activity. Despite variations in datasets depending on cell type, FORECasT-BE demonstrated predictive accuracy comparable to that of BE-Hive^84^ and DeepBaseEditor^85^ across various high-throughput cellular contexts.

C-to-G base editors (CGBEs)^90,91^ are an alternative base editing tool that induces C-to-G transversion mutations, whereas traditional base editors, such as CBEs and ABEs, primarily induce transition mutations. Koblan et al.^92^ generated an additional dataset by characterizing 10 CGBE variants (various CBE derivatives, lacking a uracil DNA glycosylase inhibitor or incorporating DNA-repair proteins) at more than 10,000 target sites in mammalian cells. These data were used to train a model similar to the traditional BE-Hive^84^, leading to the development of the CGBE-Hive model^92^, which predicts the purity and yield of editing outcomes for CGBEs. Using CGBE-Hive, they designed CGBEs and their gRNAs to introduce desired edits, successfully correcting the amino acid sequences of 546 disease-related SNVs with over 90% accuracy.

Moreover, researchers have expanded the scope by modeling a large number of base editor variants. Kim et al.^93^ devised DeepCas9variants and DeepBE, which predict the base editing activity and outcomes of 63 base editors, formed by combining nine PAM-compatible Cas9 variants and seven deaminase variants including CBEs, ABEs and CGBEs. They conducted a DL-based computational model using three sublibraries consisting of a total of 47,475 pairs of gRNAs and targets. When tested on three base editors not included in the training datasets, DeepBE achieved an average Pearson correlation value of 0.77, demonstrating high performance.

### Strengthening base editing predictions through endogenous target analysis

Most studies have relied on synthetic environments to generate high-throughput data for training models. However, artificial target sites using lentiviral integration often overestimate the editing activity or exhibit poor correlation with editing outcomes at some endogenous targets. Li et al.^94^ designed an automated platform for genome editing at 1210 endogenous target sites. Based on results from in situ genome engineering, they generated the chromatin accessibility enabled learning model (CAELM), which integrates chromatin accessibility and sequence context to predict the outcomes of CBE (BE4max, AncBE4max and hyA3A-BE4max)^86^. In a comparable approach, Yuan et al.^95^ obtained a genome-wide dataset from about 5000 endogenous target sites to address these discrepancies. By analyzing data, they discovered that base editing of ABEmax-F148A or YE1-BE3-FNLS is affected by endogenous factors such as transcriptional activity, chromatin accessibility, and DNA and histone modifications at endogenous target sites. The researchers also developed a DL algorithm called BE_Endo, which incorporates endogenous factors and sequence information. Because CBE activity is more likely to be influenced by endogenous factors than ABE, BE_Endo shows better predictive performance for CBE.

### Minimizing off-target effects in CBE and ABE

The genome contains numerous sequences that are similar to the target site where gRNAs can bind. As base editors utilize DNA-binding properties of CRISPR, they can induce genome-wide off-target effects^96,97^, similar to CRISPR nucleases. In addition, the deaminase enzyme in base editors may exhibit CRISPR-independent off-target activity^98^. Therefore, predicting off-target effects of base editors and understanding the mismatch tolerance between gRNAs and target DNA is crucial. Zhang et al.^99^ designed gRNA off-target pairs for ABE and CBE, generating off-target efficiency datasets comprising 54,663 and 55,727 entries in human cells, respectively (Fig. 2 and Table 3). Leveraging these data, they developed DL models, ABEdeepoff and CBEdeepoff (BEdeepoff), to predict off-target sites at endogenous loci. These tools can help to reduce the off-target effects associated with base editing.

### Discovering enhanced cytidine deaminases for base editing

Huang et al.^100^ used AlphaFold2 to model the structure of 238 protein sequences from various deaminase families and found many ssDNA and dsDNA cytidine deaminases by clustering them on the basis of structural similarities. They further applied AI-assisted structural prediction to minimize the size of ssDNA deaminase (Sdd). Using the miniaturized Sdd, they created a CBE that could be packaged into a single vector for AAV-based CRISPR–Cas9 base editing. Likewise, Xu et al.^101^ conducted both amino acid homology searches and structure-based similarity analysis using the three-dimensional structures generated by AlphaFold2. A total of 1483 APOBEC-like deaminases were identified through amino acid homology analysis, and they were classified into 184 clusters using AI-based structural prediction. The study uncovered deaminases with high editing efficiency and robust activity across diverse sequence contexts. In another study, multiple synthetic adenine deaminases were discovered from a TadA-like sequence dataset using ProGen2^82^ and ProteinMPNN^102^, just as OpenCRISPR-1 was identified from a Cas9-like sequence dataset^81^. The newly engineered adenine deaminases, when fused to SpCas9 or OpenCRISPR-1 to create ABEs, demonstrated robust A-to-G editing. These findings suggest that AI could substantially broaden the scope of base editor applications (Fig. 2).

## Elevating CRISPR prime editing with AI

Prime editing is a gene-editing technology that allows more diverse edits compared with CRISPR nuclease and base editing. The prime editor^21^ used in prime editing consists of two main components: a protein component, which is a nickase Cas9 fused to a reverse transcriptase, and the prime editing gRNA (pegRNA), which is extended from gRNA with a primer binding site (PBS) and a reverse transcription template (RTT). Once the spacer sequence of the pegRNA binds to the target site, the nickase Cas9 generates a single-strand break on the non-target DNA. After nicking, a segment of ssDNA is exposed near the cut site, providing a substrate for the reverse transcriptase. This exposed ssDNA anneals to the PBS sequence of the pegRNA to initiate the reverse transcription. The reverse transcriptase then synthesizes a DNA, complementary to the RTT sequence, using the pegRNA as a template. This newly synthesized DNA contains information for the intended edit and replaces the original DNA sequence through intracellular DNA repair mechanisms. Through this mechanism^103^, prime editing mediates substitutions, small insertions and deletions at the target sites without generating DSBs^21,104,105^ (Fig. 1). However, the relatively low editing efficiency remains a drawback that needs improvement. DL and ML models can accurately predict the efficiency of prime editors by leveraging large experimental datasets. In particular, by utilizing AI models in the optimization process of pegRNAs, experimental attempts can be reduced, saving both time and costs (Fig. 2 and Table 4).

**Table 4:** Prime editor system-associated AI prediction model.

Name	Prime editing system	Organism	AI-model used	Purpose	Reference (year)	Web tool or code link
DeepPE	PE2	Human	MLP	Prime editing outcome prediction	^106^ (2021)	https://deepcrispr.info/DeepPE/
Easy-Prime	PE2, PE3	Human	XGBoost	Design optimization of pegRNA	^107^ (2021)	http://easy-prime.cc/.
PRIDICT	PE2	Human	RNN (AttnBiRNN)	Prime editing outcome prediction, design optimization of pegRNA	^108^ (2023)	https://www.pridict.it/
DeepPrime, DeepPrime-Off	PE2, PE4	Human, mouse	CNN, GRU	Prime editing outcome prediction, off-target prediction	^109^ (2023)	http://deepcrispr.info/DeepPrime
OPED	PE2, PE3, ePE	Human	Transformer	Prime editing outcome prediction, design optimization of pegRNA	^110^ (2023)	http://bicdb.ncpsb.org.cn/OPED/
MinsePIE	PE2, PE3	Human	XGBoost	Prime editing outcome prediction	^111^ (2023)	https://elixir.ut.ee/minsepie/
PRIDICT2.0	PE2	Human, mouse	RNN (AttnBiRNN)	Prime editing outcome prediction	^113^ (2024)	www.pridict.it
ePRIDICT	PE2	Human, mouse	XGBoost	Prime editing outcome prediction	^113^ (2024)	www.pridict.it

### Optimizing pegRNA design for prime editing

The various versions of prime editors differ in their efficiency and accuracy, making the selection of the appropriate version and the prediction of its efficiency a critical challenge in prime editing. In response to this hurdle, multiple research teams have focused on using AI to predict prime editing efficiency (Fig. 2 and Table 4).

Kim et al.^106^ evaluated the efficiency of prime editor 2 (PE2) across various sequences and genomic contexts using 54,836 combinations of pegRNAs and target sequences. They analyzed factors that affect PE2 efficiency, such as the length of the pegRNA, including PBS and RTT regions, and the GC content of the target sequence. Based on these data, the authors established a computational model, DeepPE, to predict PE2 efficiency. In a different study, Easy-Prime^107^ was developed to optimize pegRNA and an additional nicking gRNA. The nicking gRNA improves PE activity by specifically cleaving nonedited alleles, thereby increasing the overall efficiency of prime editing, an approach referred to as the PE3 system. Researchers assessed the features affecting PE efficiency and found that the RNA folding feature was critical.

With a different large dataset using self-targeting pegRNA libraries, PRIDICT^108^ was created to predict the efficiency of PE and expected editing outcomes. Training from the data with 92,423 pegRNAs for targeting 13,349 mutations, PRIDICT provides potential pegRNAs with PRIDICT scores, based on their editing efficiencies and unintended editing rates. In another study, Yu et al.^109^ tested prime editing efficiency on 338,996 pegRNA pairs, including 3979 engineered pegRNAs and target sequences. Based on these datasets, they devised computational models called DeepPrime and DeepPrime-FT to predict the editing efficiency of all types of edits, up to three base pairs, across seven cell types and eight prime editing systems. The researchers also formulated DeepPrime-OFF to predict editing efficiency at mismatched targets, which is useful for reducing the off-target effects.

By incorporating transfer learning, an optimized prime editing design (OPED)^110^ was generated to predict efficiency and optimize the design of pegRNA. This model improves both the accuracy and generalizability of predicting pegRNA efficiency. OPED demonstrated the successful introduction of various ClinVar pathogenic mutations with PE2, PE3/PE3b and ePE systems. In addition, the authors offer OPEDVar database, a web application with optimized PE designs for over two billion pathogenic variant candidates.

Focusing on short sequence insertions among the editing outcomes of prime editors, the MinsePIE (modeling insertion efficiency for prime insertion experiments) algorithm^111^ was developed to predict insertion efficiency. This model analyzed 3604 pegRNAs across various cell lines and genomic sites, identifying key factors for efficient PE activity, such as sequence length, cytosine content and secondary structure, as well as DNA repair mechanisms such as TREX1 and TREX2. The authors also showed that MinsePIE aids in selecting codon variants of common fusion tags that achieve high insertion efficiency.

### Enhancing prime editing efficiency in chromatin contexts

The efficiency of prime editing can vary depending on the genomic environment, such as the chromatin state and epigenetic modifications surrounding the target gene. One of the most well-known cellular factors that substantially influence prime editing is the mismatch repair (MMR) pathway. The MMR pathway is a DNA repair mechanism that recognizes and corrects mismatches in the DNA^112^. During prime editing, the MMR pathway may identify the newly reverse transcribed DNA introduced by the prime editor as an error and remove it, thereby reducing the overall efficiency of prime editing. To address these factors, the PRIDICT^108^ model was further refined into PRIDICT2.0 and ePRIDICT^113^, enhancing the accuracy of prime editing efficiency predictions. PRIDICT2.0 was trained using datasets from different cell lines and contexts, such as MMR-proficient and MMR-deficient cells, allowing predictions across diverse genomic conditions. It can predict editing outcomes, including insertions, deletions and substitutions, for edits up to 15 base pairs in length. Furthermore, ePRIDICT expanded on PRIDICT2.0 by integrating local chromatin features. This model specifically accounts for the chromatin environment, which affects the efficiency of prime editing. It is particularly useful for predicting editing efficiency in different chromatin states, such as active transcription regions or heterochromatin, helping researchers to customize their approach on the basis of the genomic environment.

### Boosting prime editing efficiency with additional factors

As the MMR pathway can limit the success of prime editing by removing DNA containing the desired edits, inhibiting the MMR pathway helps to achieve higher prime editing activity^114–116^. Prime editing efficiency was improved through the key MMR pathway protein MLH1 by expressing a dominant-negative form (MLH1dn), a strategy known as the PE4 system^114^. To approach a similar strategy in a novel way, Park et al.^117^ used AI to generate a new small binder protein that binds to MLH1 and inhibits the MMR pathway. The authors utilized RFdiffusion^118^ and AlphaFold3 to design this small binder protein (MLH1-SB), and by incorporating it into the prime editor, they observed an improvement in prime editing efficiency. By using AI-assisted protein engineering to create new elements that can boost genome editing efficiency, a much wider variety of genome editing tools will emerge.

## Conclusion

The convergence of AI and CRISPR technology has massively increased activity and specificity in genome engineering. AI technology, which excels at analyzing and recognizing features and patterns from large amounts of experimental data, predicts the efficiency and accuracy of gene editing. Computational tools can quickly perform complex calculations by searching for systems based on amino acid sequences or protein structural homology, identifying unknown CRISPR-like systems from vast amounts of genomic information. In addition, AI-driven protein design models are used to design new proteins for genome engineering that do not exist in nature. AI technology plays an important role in reducing the number of experiments by computationally suggesting the most optimal pathway, reducing the probability of failure. In this Review, we have outlined that CRISPR-mediated genome engineering can be improved and uncovered using AI tools.

Nevertheless, current technical limitations constrain the full potential of AI. The performance of AI models is highly dependent on the conditions and quality of the experimental dataset. If the specific conditions of an organism, tissue or cell type differ, the predictions made by ML may not match the experimental results. In addition, while generative AI enables the design of new proteins, the original data on which they are trained are derived from existing proteins in nature; therefore, they cannot suggest brand-new designs. There may also be missing information about the properties of the protein, and the functionality of the AI-designed protein needs to be validated. However, AI technology is evolving at an accelerated pace; thus, these problems will be solved in the near future.

With the recent clinical approvals of gene-editing therapies, genome engineering technologies become critical in their ability to directly correct the DNA mutations that cause rare genetic diseases^17^. In therapeutic applications, CRISPR technologies must achieve greater precision, high efficiency and safety, not only at the cellular level but also within the human body. AI-enhanced CRISPR activity prediction and functional improvements will help to develop personalized treatments for each patient, considering the complexity of their individual genomes. By effectively applying AI, researchers can develop safer and more precise CRISPR tools for clinical use, leading the way forward in gene therapy.

Consequently, AI decreases the burden on researchers in genome engineering by improving existing CRISPR technologies and making it possible to create new systems. By learning from the extensive experimental data in genome editing, along with predictive modeling, AI will continue to elevate the efficiency, accuracy and safety of CRISPR technologies. As AI-driven CRISPR technologies become more sophisticated, they will accelerate the development of next-generation personalized therapies for genetic disorders, bringing a new era of precision medicine.
